# Synergy of osmotic adjustment and antioxidant activity in *Catalpa bungei*: alleviating persistent drought stress from SL to NSL

**DOI:** 10.3389/fpls.2025.1536795

**Published:** 2025-03-12

**Authors:** Xiaochi Yu, Junhui Wang, Wenjun Ma, Fei Yi, Peng Zhang

**Affiliations:** ^1^ Forestry College, Northeast Forestry University, Harbin, China; ^2^ State Key Laboratory of Tree Genetics and Breeding, Research Institute of Forestry, Chinese Academy of Forestry, Beijing, China; ^3^ College of Biological and Pharmaceutical Sciences, Three Gorges University, Yichang, China

**Keywords:** *Catalpa bungei*, drought stress, stomatal limitation, non-stomatal limitation, synergistic regulation

## Abstract

**Introduction:**

*Catalpa bungei* C. A. Mey is a precious timber and garden tree species native to China. It is mainly distributed in the semi-arid regions of northern China, where drought stress severely affects its growth.

**Methods:**

In this study, we investigated the physiological responses and gene expression profiles of *C. bungei* seedlings subjected to a 28-day drought stress treatment.

**Results and discussion:**

By reducing stomatal conductance (Cond) and increasing proline (Pro) and soluble sugar contents (SS), *C. bungei* alleviated mild drought stress (7-14 days). Under moderate drought stress (14-21 days), a synergistic interaction of jasmonic acid (JA) and abscisic acid (ABA) enhanced catalase (CAT) activity and proline (Pro) content, while downregulating guard cell osmotic potential, thereby further decreasing stomatal conductance (Cond). Upon reaching severe drought stress (21-28 days, SWC 22%, LWC 73%), the activity of antioxidant enzymes and the content of osmotic substances continued to increase, while the structure of photosynthetic organs was damaged, resulting in a shift from stomatal limitation (SL) to non-stomatal limitation (NSL). Therefore, *C. bungei* mitigates mild drought stress through osmotic regulation, and ABA and JA coordinate antioxidant defenses and osmotic regulation as drought persists. Once the shift from SL to NSL caused by severe drought stress, the aforementioned mechanism ceases to be effective in mitigating the deleterious effects of drought stress on *C. bungei*. These findings enhance our comprehension of the mechanisms underlying *C. bungei*'s response to prolonged drought, providing valuable insights for the precise evaluation of drought intensity and facilitating efficient management of *C. bungei* plantations.

## 1 Introduction

Approximately one-third of the world's land is situated in arid and semi-arid regions, and the severity and frequency of drought continue to increase (Edenhofer et al., 2014). Drought reduces plant photosynthetic efficiency, restricts normal growth and metabolism, and can even lead to plant mortality (Sanders and Arndt, 2012; Oswald and Aubrey, 2024). Consequently, frequent and prolonged droughts have become one of the primary environmental factors limiting the growth, distribution, and survival of trees worldwide (Ambrose et al., 2015).

Drought stress is a dynamic development process, and the extent of its impact on plants depends on the duration and intensity of the stress (DaCosta and Huang, 2007). Under mild to moderate drought stress, the reduction in stomatal aperture leading to decreased photosynthetic rates is referred to as stomatal limitation (SL) (Buckley and Mott, 2013; Cai et al., 2015). At this stage, once drought stress is alleviated and stomata reopen, the plant’s photosynthetic capacity can return to its original levels (Flexas et al., 2009; Campos et al., 2014). Severe drought, however, causes irreversible damage to the photoresponsive system and photosynthetic organs, leading to a decline in photosynthetic capacity due to non-stomatal limitation (NSL) (Signarbieux and Feller, 2011; Liang et al., 2019). Consequently, the shift from SL to NSL is a key indicator of the transition from mild to severe drought stress in plants (Bacelar et al., 2009), marking a critical turning point from environmental constraints to physiological limitations (Song et al., 2020; Pilon et al., 2018). Leaf water content plays a clear indicative role in this shift from SL to NSL, serving as an important indicator for measuring the severity of drought stress in plants (Ullah et al., 2013; Song et al., 2020).

Under drought stress, plants lower their osmotic potential and maintain cellular homeostasis by increasing the accumulation of intracellular free proline (Pro), betaine, and soluble sugars (SS) (Blum, 2017; Zeng et al., 2015). Increasing the activity of antioxidant enzymes such as superoxide dismutase (SOD), peroxidase (POD), catalase (CAT) helps balance the level of intracellular reactive oxygen species (ROS) and reduce the accumulation of harmful substances (Saxena et al., 2016). In addition, plant hormones like abscisic acid (ABA) and jasmonic acid (JA) can also improve drought tolerance by regulating stomatal aperture (Li et al., 2020; Wang et al., 2020) and activating the expression of antioxidant enzyme genes (Macková et al., 2013). As a critical marker of the aggravation of drought stress, the process of SL to NSL transformation requires special attention in the study of plant drought resistance mechanisms, including how plants respond and coordinate these regulatory mechanisms.

*Catalpa bungei* C. A. Mey, a tree species belonging to the genus *Catalpa* in the Bignoniaceae family, is known for its high-quality timber and has a wide range of applications (Jing et al., 2015; Wang et al., 2016; Zheng et al., 2017). *C. bungei* is predominantly distributed in the semi-arid regions of northern China, where it is significantly affected by drought (Wang et al., 2016). In this study, a 28-day greenhouse drought experiment was 9conducted to determine the leaf water content (LWC) threshold at which the photosynthetic process of *C. bungei* shifts from SL to NSL. The dynamic responses of antioxidant enzyme activity, osmotic regulatory substances, and hormone levels were also analyzed. This study will explore the adaptive mechanisms of *C. bungei* under prolonged drought stress, providing guidance for accurately assessing drought damage and promoting efficient production in plantation forests.

## 2 Materials and methods

### 2.1 Plant cultivation and treatments

The experiment was conducted in the greenhouse of the Chinese Academy of Forestry (Beijing, China, 40°0′27′′ N, 116°15′22′′ E), a glass-enclosed greenhouse. The tissue cultured clone of *Catalpa bungei* (clone ID, “2-8”) were rooted and planted in pots (one plant in each pot) with sand and soil (V_sand_ : V_siol_, 1:1), which were cultivated in the greenhouse (light: natural light; temperature during the day: 24 ± 2 °C; temperature at night: 18 ± 2 °C; relative humidity: 60 ± 5%). In March of the subsequent year, the plants were pruned and transferred to large pots. They were irrigated with distilled water every three days. And 5 g slow-release fertilizer (Plantosan N-P-K 20-10-15,contains 6% Mg, 2% S, and trace elements including B、Cu、Fe、Mn、Mo and Zn;Manna, Düsseldorf, Germany) are mixed into each potting substrate. Plants (60 plants in total) with similar growth performance were selected for further experiments.

Before the start of drought treatment, all seedlings should be thoroughly watered, and the soil moisture level (SWL) after watering is approximately 53%. From July 12, all seedlings should not be watered, and at each time point of 0, 7, 14, 21, and 28 days, six plants were selected for measuring photosynthetic parameters, followed by harvesting for subsequent analysis.

### 2.2 Harvesting

Gas exchange and PSII photochemistry were analyzed before each harvesting. At each time point, six plants were selected (n=6), and for each plant, 3 leaves from the 3rd to 5th healthy functional leaves from top to bottom for each seedling were selected, selected to measure photosynthetic rates (Pn), stomatal conductance (Cond), intercellular CO_2_ concentration (Ci) and transpiration rate (Tr) by a portable photosynthesis system (Li-6400, licor Biosciences, Lincoln, NE, USA) and LI-6400XT red and blue light source. The measurements were carried out from 09:00 to 11:00 h. During the measurement of gas exchange, the photosynthetic photon flux density (PPFD) was set at 1200 µmol·m^-2^·s^-1^, CO_2_ levels at 400 ppm, block temperature at 30 °C, and relative humidity (RH) was 44.7%. The stomatal limitation value (Ls) was calculated according to Formula 1:


(1) Ls=1-Ci/Ca(atmospheric carbon dioxide concentration)

The photosynthetic light response curves were measured on three healthy functional leaves from the 3rd to 5th positions of each of the six seedlings mentioned above, using the Handy PEA (Version 1.0, Hansatech Instruments Ltd. Norfolk,UK) between 9:00 and 11:00. After dark adaptation of plant leaves for 30 minutes, *Fo* (initial fluorescence), *Fv* (variable fluorescence), and *Fm* (maximum fluorescence) were measured, and *Fv*/*Fo* (potential activity of PS II), *Fv*/*Fm* (maximum photochemical efficiency of PS II), and PI (photo retention index) were calculated, the calculation method is based on Formula 2:


(2) PI=(Saturated light intensity-Fo)/Saturated light intensity

After the observation of photosynthetic physiological observations on the six plants, three leaves were separated from the plants by scissors and soil from the pot were got. The fresh weight was obtained and then the leaves and soil were put into separate paper bag, then put into the oven for 105 °C deactivation for 1 h, then dried to constant weight at 80 °C to determine the dry weight. The leaf water content (LWC) and soil water content (SWC) were calculated by the Formulas 3, 4:


(3) $$\begin{array}{c} \text{LWC}=\left(\text{Leaf fresh weight}-\text{leaf dry weight}\right)/\text{leaf fresh weight}\\ \times 100\% \end{array}$$

(4) $$\begin{array}{c} \text{SWC}=\left(\text{Soil fresh weight}-\text{soil dry weight}\right)/\text{soil fresh weight}\\ \times 100\% \end{array}$$

### 2.3 Determination of enzymatic activities, proline, soluble sugars and phytohormoens

At each time point, six plants were selected for physiological indicator measurements and transcriptional level analysis. Superoxide Dismutase (SOD; EC l.15.1.1) activity was measured determined according to the method of Cao et al (2014). The peroxidase (POD; EC 1.11.1.7) and catalase (CAT; EC 1.11.1.6) activity were measured by the nitroblue tetrazolium method (NBT), the guaiacol method, respectively (Shi et al., 2017). The soluble sugar content (SS) and free proline content (Pro) in leaves were measured by anthrone colorimetry and spectrophotometry (McDowell, 2011). Jasmonic acid (JA) and abscisic acid (ABA) in the leaves were measured by high-per-formance liquid chromatography-electrospray ionization-ion trap mass spectrometry (Shi et al., 2015).

### 2.4 Analysis of transcript levels of genes involved in drought resistance

The transcriptional levels of genes participated in drought resistance were analyzed by qPCR based on Cao 's method (Cao et al., 2014). According to the reported studies (Cao et al., 2014; Shi et al., 2017), genes encoding the biosynthetic enzymes such as 12-oxophytodienoic acid 10,10-reductase (OPR) and 9-cis-epoxycarotenoid dioxygenase (NCED) were selected for transcriptional analysis. There are two pathways for synthesizing proline in higher plants, so the encoding genes for key enzymes delta-1-pyrroline-5-carboxylate synthase (P5CS) and ornithine-delta-aminotransferase (OAT) in both pathways were used for transcriptional analysis. Transcript change of genes encoding POD, SOD, CAT and GPX were also analyzed as they function as ROS scavenger. Sucrose and trehalose play important roles in regulating plant cell osmotic pressure, and their contents are regulated by sucrose synthase (SuS) and trehalose phosphate synthase (TPS), respectively. Therefore, the transcription levels of their encoded genes were measured during different drought stress periods.

### 2.5 Statistical analysis

The data were checked for normality before statistical analysis. Drought was set as experimental factors. All data were tested using the one-way ANOVA procedure in SPSS (version 22.0). Multiple mean comparisons (Duncan test) were applied. Differences were considered to be significant if the *p value* was less than 0.05. The figures include bar charts, four-parameter fitting curves and second order polynomial were prepared in GraphPad Prism (version 7).

## 3 Results

### 3.1 Gas exchange parameters and PSII photochemistry

**Figure 1:**
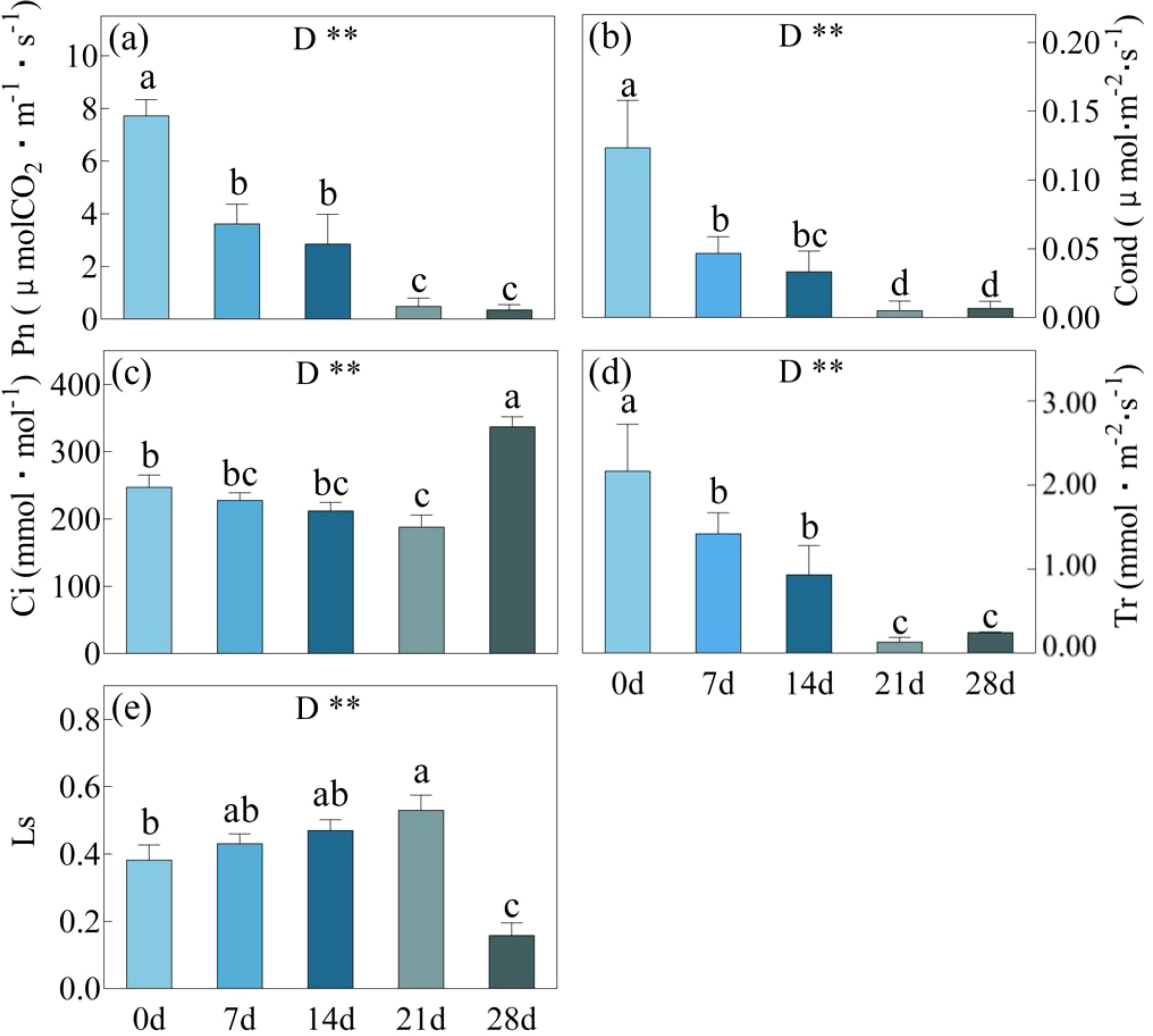
Pn, Photosynthetic rate **(a)**; Cond, Stomatal conductance **(b)**; Ci, Intercellular CO2 concentration **(c)**, Tr, Transpiration rate **(d)** and Ls, stomatal limitation value **(e)** in *C. bungei* under drought stress for 0, 7, 14, 21 and 28 days. Error bars represent the SD received from five biological replicates. Statistical analysis was performed using Duncan’s multiplication range test. *P*-values obtained from the one-way ANOVAs for drought stress. **p< 0.01. the same applies below. Different letters on the bars indicate significant differences between the five treatments based on Duncan’s test (P<0.05). The same applies below.

Drought stress was gradually induced by withholding water for 0, 7, 14, 21 and 28 days in *C. bungei*. After day 7 and day 21 of drought, there were significant downward trends observed in Pn, Cond, and Tr (**Figure 1**). However, there were no significant differences in these indicators between the day 21 and day 28 drought periods. As the duration of drought stress continued to increase, there was a gradual but non-significant decrease in Ci and an increase in Ls, and this trend persisted until day 21. However, after the day 28, there was a significant increase in Ci and a remarkable decrease in Ls, compared to the conditions observed at day 21.

**Figure 2:**
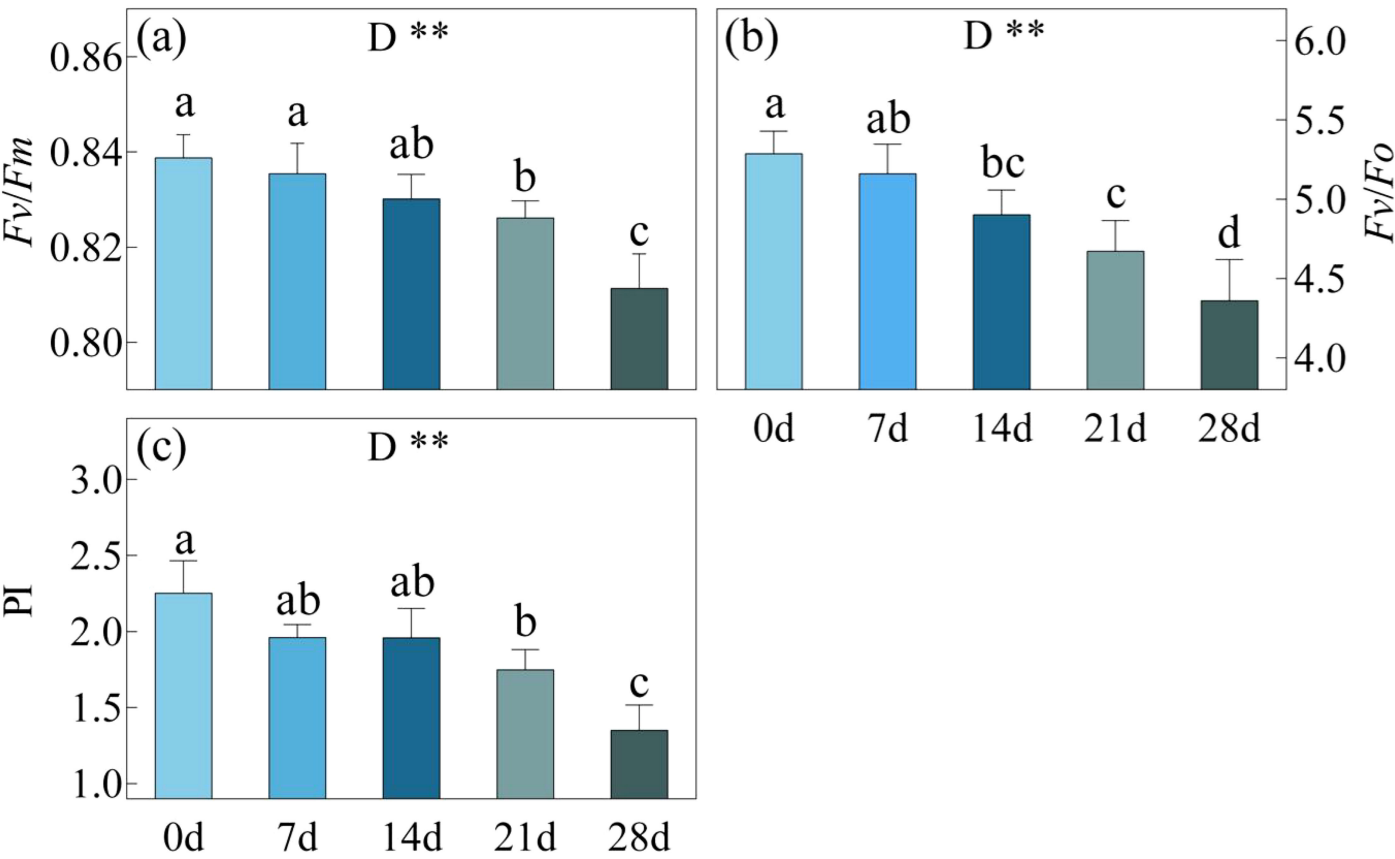
*Fv*/*Fm*, Maximum quantum yield of photosystem II **(a)**, *Fv*/*Fo*, Efficiency of the water-splitting complex on the donor side of PSII **(b)** and PI, the performance index total **(c)** in *C. bungei* under drought stress for 0, 7, 14, 21 and 28 days. P-values obtained from the one-way ANOVAs for drought stress. **p< 0.01.

With the continuation of drought stress, there was a gradual decline in the values of *Fv*/*Fm*, *Fv*/*Fo* and PI (**Figure 2**). By the day 21 of drought, *Fv*/*Fm*, *Fv*/*Fo* and PI decreased by ca. 1.5%, 11.6%, 7.5% and 22.3% respectively compared to day 0. However, by the day 28, these values exhibited a significant drop compared to the day 21, even surpassing the day 0 with reductions of ca. 3.3%, 17.5%, 20.6% and 40.0%.

### 3.2 Soil moisture content and leaf water content

**Figure 3:**
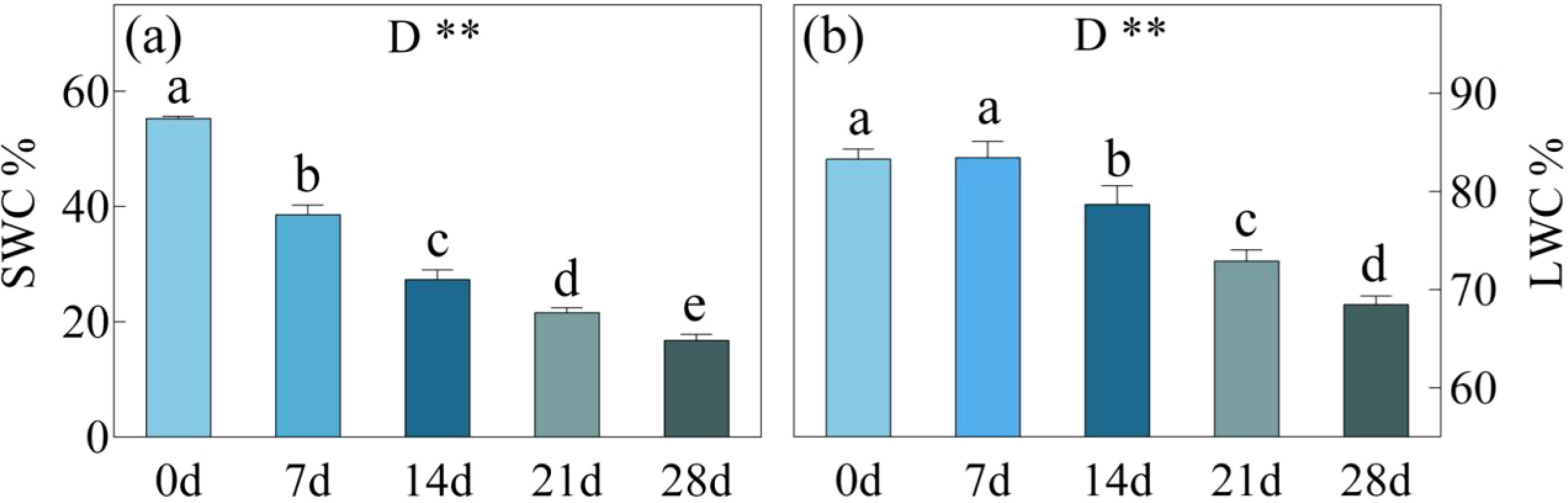
SWC, soil moisture content **(a)**; LWC, Leaf water content **(b)** in *C. bungei* under drought stress for 0, 7, 14, 21 and 28 days. P-values obtained from the one-way ANOVAs for drought stress. **p< 0.01.

**Figure 4:**
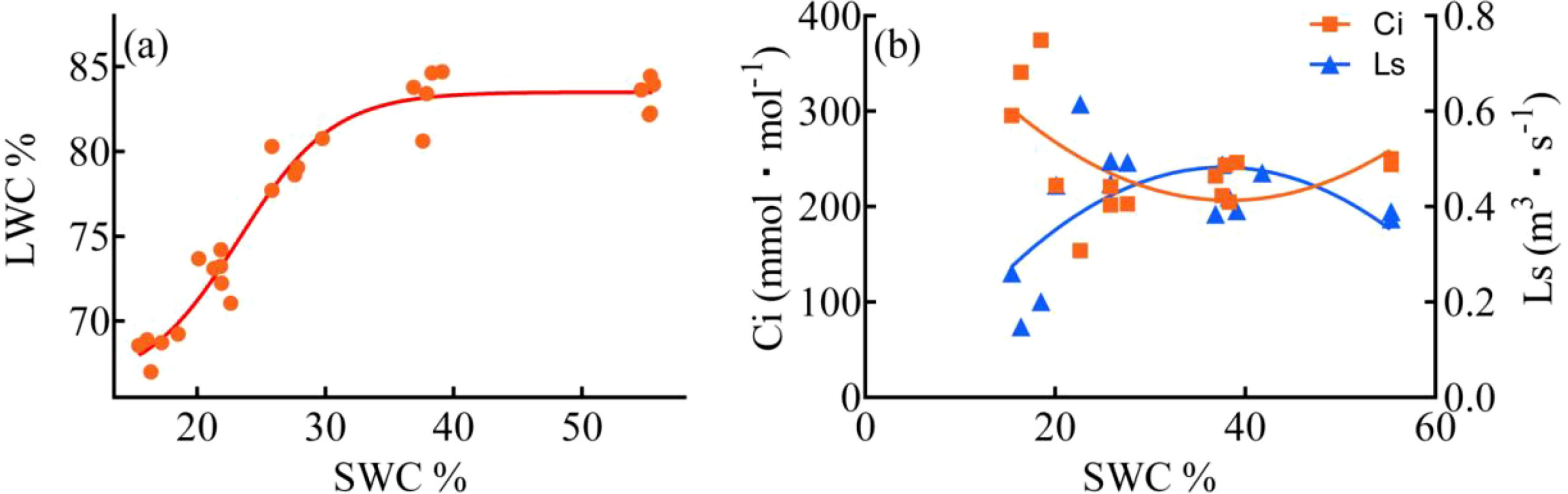
Four-parameter fitting curves for LWC (Leaf water content) **(a)**, and SMC (soil moisture content) **(b)**, Second order polynomial for Ci and LWC, Ls and LWC.

As the duration of drought stress increased, there was a substantial decline in SMC. Over the course of 7, 14, 21, and 28 days of drought stress, SMC decreased by ca. 30%, 50%, 61%, and 70% respectively, compared to 0 day (**Figure 3**). The reduction in SMC became progressively slower with longer durations of drought stress. The LWC was almost unaffected by a 7-day drought stress, but after day 14, 21, and 28, the LWC decreased by ca. 5%, 12%, and 18% respectively, compared to day 0. The analysis of the four-parameter curve fitting for LWC and SMC revealed interesting findings (**Figure 4a**). During the 0-7 days and 7-14 days of the drought treatment, there was a gradual decline in LWC, with the slope of the leaf/soil water content curve being less than 0.40. However, after day 14, when the SWC dropped below approximately 30%, there was a significant decrease in LWC. The slopes of the leaf/soil water content curve during the 14-21 days and 21-28 days period reached 0.97 and 0.78, respectively, indicating a pronounced reduction in LWC. In addition, the Ci, Ls/LWC fitting curve revealed that the points at which Ci and Ls underwent changes were situated within the range of 78% - 82% LWC (**Figure 4b**).

### 3.3 Antioxidant enzyme activity and osmotic regulators

**Figure 5:**
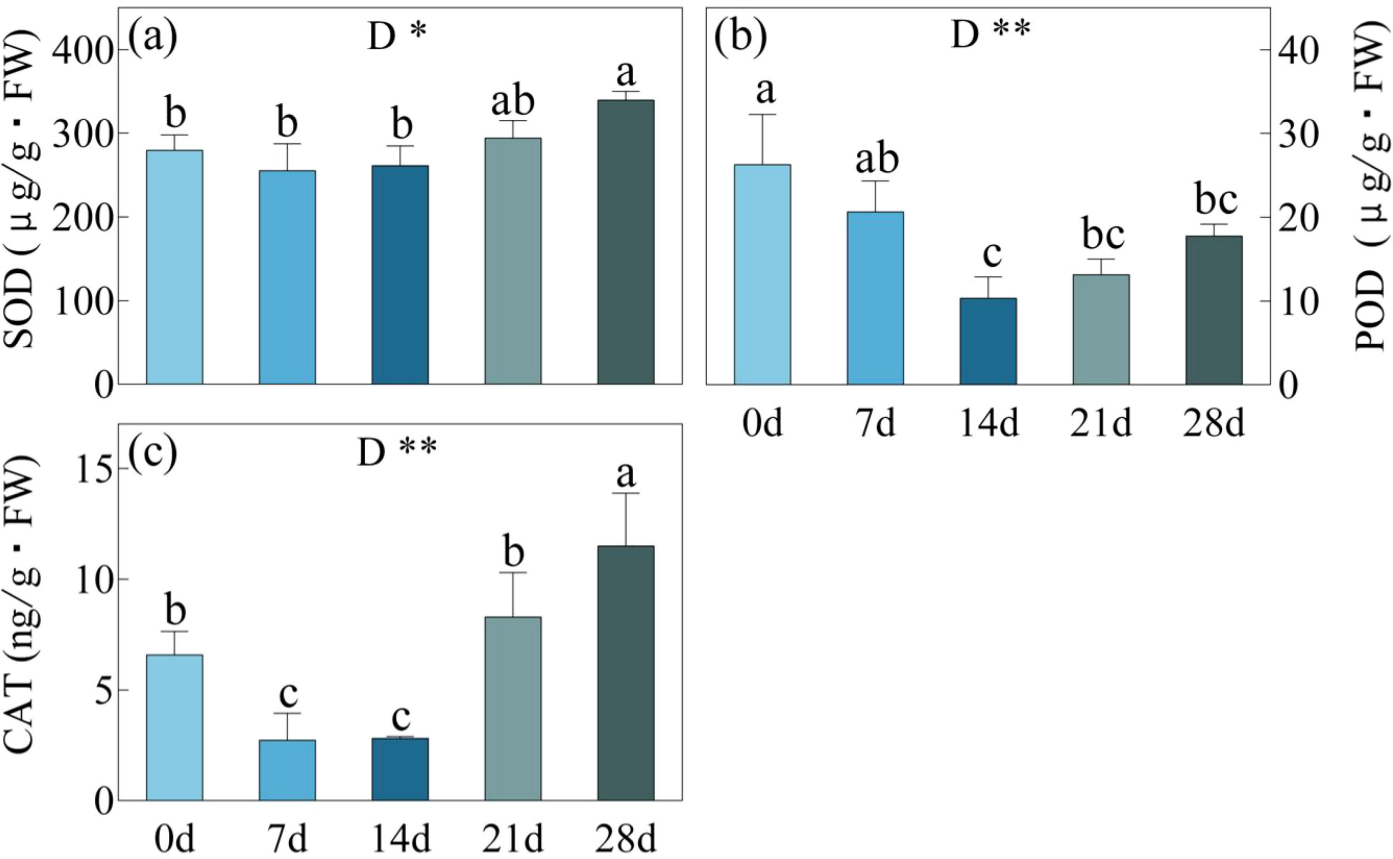
SOD, Superoxide Dismutase enzyme activity **(a)**; POD, Peroxidase enzyme activity **(b)** and CAT, Catalase enzyme activity **(C)** in *C. bungei* under drought stress for 0, 7, 14, 21 and 28 days. *p< 0.05. the same applies below. P-values obtained from the one-way ANOVAs for drought stress. **p< 0.01.

During the 7-day and 14-day drought stress periods, SOD activity remained unchanged, while the activities of POD and CAT decreased (**Figure 5**). The lowest POD activity was observed at day 14, pointing to 10.3 μg/g·mm, while CAT activity remained consistently low from day 7 to day 14, with values ranging from 2.7-2.8 ng/g. After day 14, the mentioned indicators started to increase. By the day 21, SOD, POD, and CAT activities reached 294.3 μg/g, 13.1 μg/g·mm, and 8.3 ng/g, respectively. On the 28th day, the activities of SOD and CAT enzymes showed further significant increases, reaching 172.89 μg/g and 17.75 ng/g. These were significantly higher than the activities observed day 0, where SOD was measured at 145.8 μg/g and CAT at 9.0 μg/g. On the other hand, although there was an increase in POD enzyme activity, it remained below the level observed initially.

**Figure 6:**
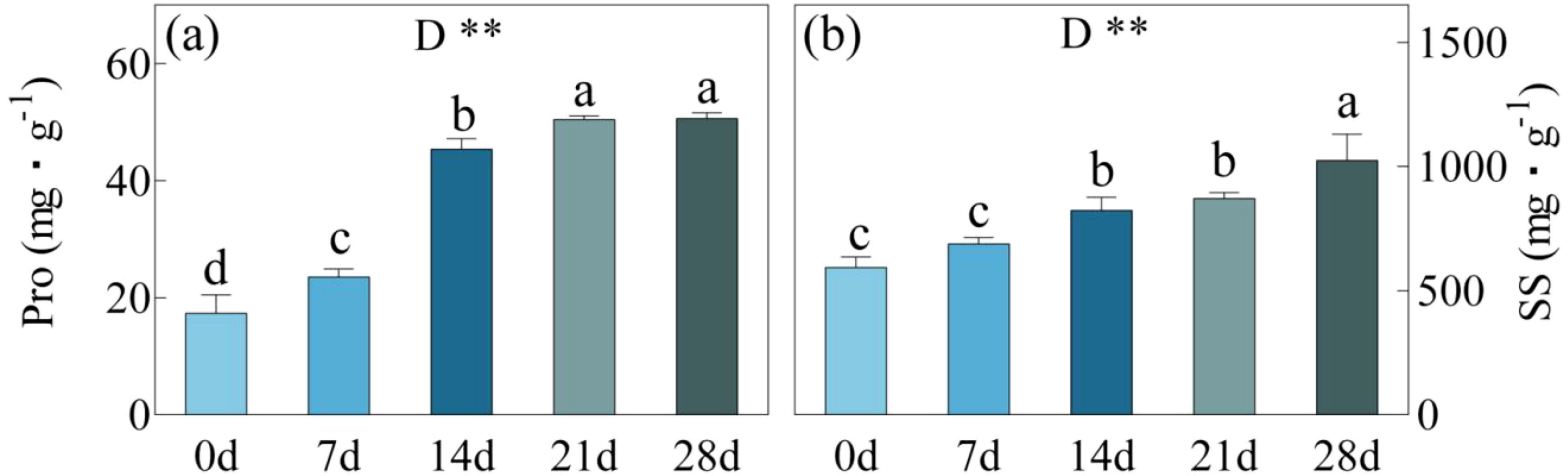
Pro, Proline content **(a)** and SS, Soluble sugar content **(b)** in *C. bungei* under drought stress for 0, 7, 14, 21 and 28 days. P-values obtained from the one-way ANOVAs for drought stress. **p< 0.01.

Pro displayed a significant rise after days 7, 14, and 21 of drought stress, surpassing day 0 by 36%, 161%, and 190% respectively (**Figure 6**). However, there was no significant variation in Pro between day 21 and 28, ranging from 50.45 to 50.61 mg/g. During the onset of drought stress, SS content exhibited a steady, incremental rise. At days 7, 14, and 21, there were improvements of 16%, 39%, and 47% compared to day 0. Furthermore, from day 21 to 28, their content significantly increased from 8716.28 mg/g to 10242.90 mg/g, representing a 73% boost after 28 days compared to day 0.

### 3.4 Hormone concentration

**Figure 7:**
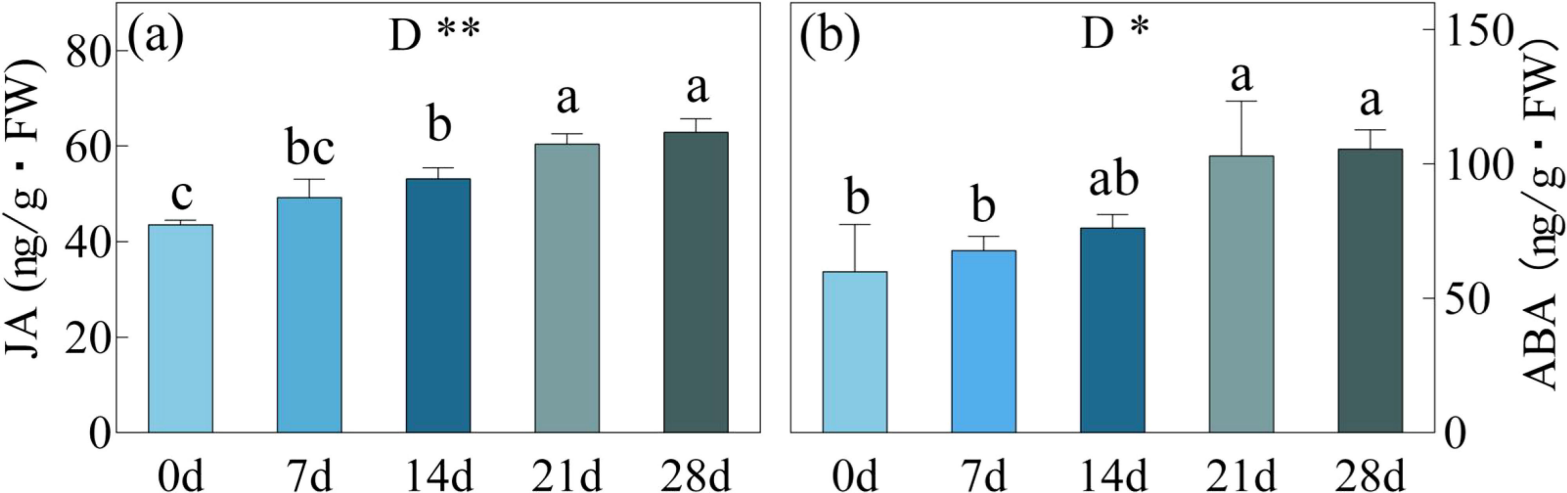
JA, Jasmonic Acid **(a)** and ABA, Abscisic Acid **(b)** in *C. bungei* under drought stress for 0, 7, 14, 21 and 28 days. P-values obtained from the one-way ANOVAs for drought stress. **p< 0.01,*p< 0.05.

When subjected to drought stress for 7 and 14 days, JA showed an increase of 13.08% and 27.07% in concentration, while ABA exhibited respective increases of 13.08% and 22.00% (**Figure 7**). Following a prolonged 21-day period of drought stress, notable spikes in JA and ABA levels were observed, reaching 102.99 ng/g and 60.45 ng/g, signifying a remarkable growth of 71.65% and 38.84% from day 0. However, during the subsequent 21 to 28-day interval, JA and ABA concentrations did not exhibit significant changes, maintaining a range of 102.99-105.59 ng/g (JA) and 60.45-62.89 ng/g (ABA) respectively.

### 3.5 Transcriptional changes of genes responsible for drought resistant

**Figure 8:**
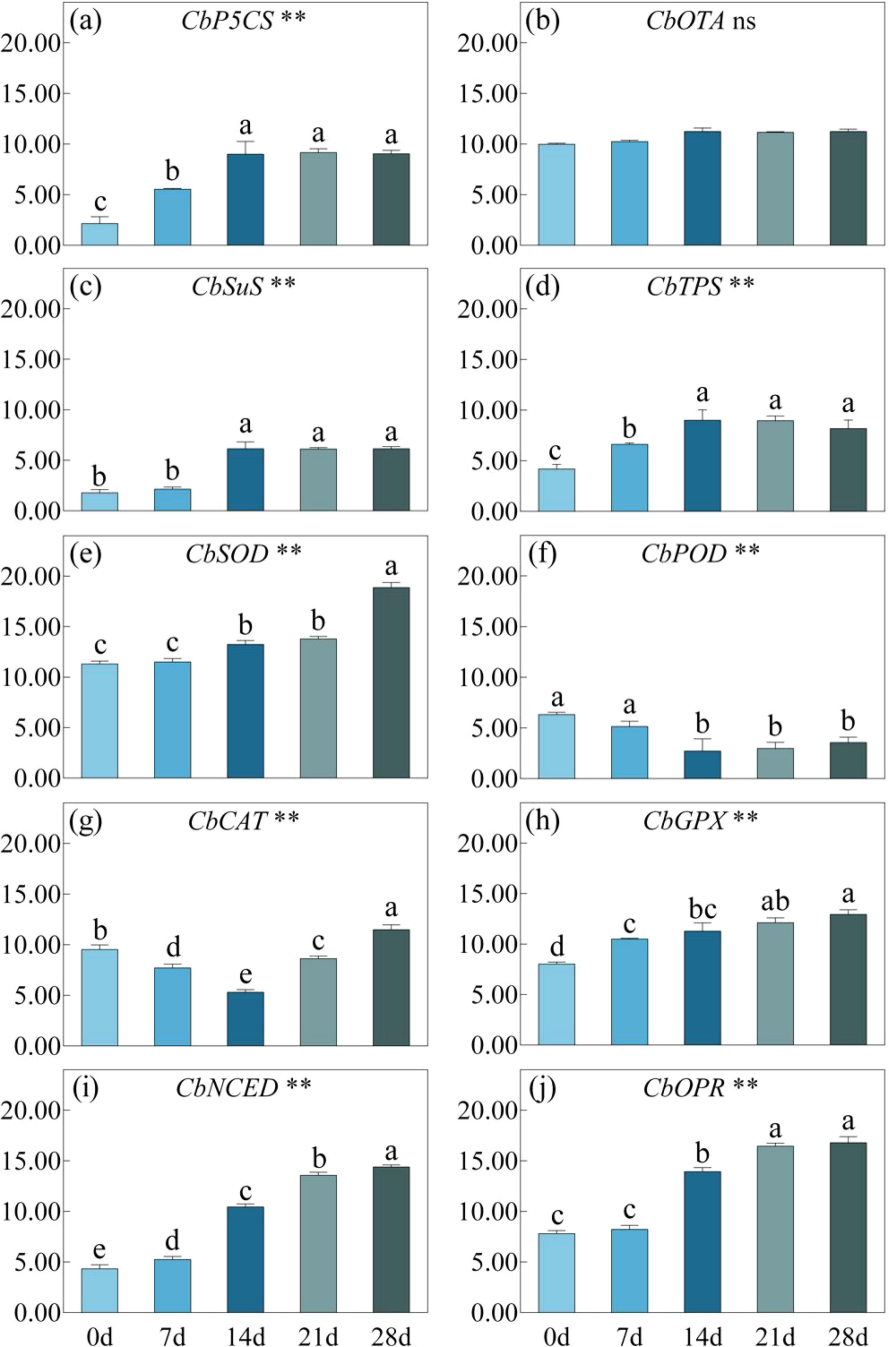
*CbP5C5* **(a)**, *CbOTA* **(b)**, *CbSuS* **(c)**, *CbTPS* **(d)**, *CbSOD* **(e)**, *CbPOD* **(f)**, *CbCAT* **(g)**, *CbGPX* **(h)**, *CbNCED* **(i)**, *CbOPR* **(j)** under drought stress for 0, 7, 14, 21 and 28 days. P-values obtained from the one-way ANOVAs for drought stress. **p< 0.01, ns p>; 0.05.

Under drought stress, the relative expression of *CbP5CS* significantly increased until 14 days and remained stable thereafter, but the expression level of *CbOAT* did not show significant changes throughout the entire treatment process (**Figure 8**). The expression trends of *CbSuS* and *CbTPS* were consistent, both reached their peak after 14 days of drought stress, and then showed no significant changes. The expression of genes encoding antioxidant enzymes varied greatly. As the drought persisted, the expression levels of *CbSOD* and *CbGPX* both increased continuously, while the expression of *CbPOD* decreased at day14 and remained at a low level thereafter. Affected by drought stress, the expression of *CbCAT* decreased and reached its lowest point at day 14, then significantly increased. The expression level of on day 28 was significantly higher than that without stress. Corresponding to the changes in ABA and JA content, the expression of *CbNCED* and *CbOPR* continued to increase and stabilized after 21 days of drought stress.

## 4 Discussion

### 4.1 Non-stomatal limitations indicate that *C. bungei* are experiencing severe drought stress

The adaptive mechanisms of plants to drought stress vary with the severity of the stress. As drought stress intensifies, the limitation on photosynthesis shifts from being stomatal to non-stomatal (Song et al., 2020). In this study, during the 0-21 day drought period, *C. bungei* exhibited a gradual decline in Pn, Cond, Ci, and Tr. As severe drought stress had not been reached (SWC > 21.6%), the *C. bungei* leaves reduced stomatal aperture to minimize water loss through transpiration, which in turn reduced the CO_2_ influx into the stomata, leading to a decrease in Ci and consequently a decline in photosynthetic rate. At this time, there was a slight, though not significant, decrease in *Fv*/*Fm*, *Fv*/*Fo*, and PI, indicating that the photosynthetic apparatus had not yet suffered significant damage (Buckley and Mott, 2013; Cai et al., 2015). During the 21-28 day treatment period, drought stress intensified further, causing the stomata of *C. bungei* leaves continued to decrease or even close. The process of CO2 entering the leaves was blocked, however, as O_2_ became the primary electron acceptor in the cells (Flexas et al., 2004), Ci increased significantly. At this point, the dynamic balance between the production and clearance of reactive oxygen species (ROS) was completely disrupted, leading to a rapid increase in oxidation rates and free radicals. This resulted in the degradation of photosynthetic pigments, damage to the chloroplast structure, and the impairment of photosynthetic organs, ultimately causing a decline in photosynthetic capacity (Signarbieux and Feller, 2011; Liang et al., 2019). Although Ci increased, there were no significant changes in Pn, Cond, and Tr between the 21 day and 28 day. In this study, one of the indicators marking the shift from SL to NSL was the abrupt increase in Ci and the sudden decrease in Ls. This finding is consistent with conclusions in plants such as maize (*Zea mays* L.) (Song et al., 2020) and sea buckthorn (*Hippophae rhamnoides* L.) (Liu et al., 2017), where changes in Ci and Ls were identified as key determinants in whether photosynthesis is limited by SL or NSL. Another indicator was the inconsistency between the changes in Ci and Cond. If Ci and Cond decrease simultaneously under stress, then the inhibition of photosynthesis is mainly caused by SL. If the changes in Ci and Cond are inconsistent, then NSL plays a dominant role. Li et al.'s study reached the same conclusion. There is also a sharp decrease in Fv/Fm, Fv/Fo, and PI. The maximum quantum yield (*Fv*/*Fm*) of photosystem (PS) II is the primary yield of PSII photochemistry and is considered a potential indicator of photosynthetic capacity (Stallmann et al., 2018). The efficiency of the water-splitting complex on the donor side of PSII (*Fv*/*Fo*) reflects the efficiency of photosynthetic electron transport, while the photosynthetic performance index (PI) represents the overall functionality of PSII (Zhang et al., 2015). When the LWC of *C. bungei* decreased to between 68.5% and 72.9%, the sharp decline in these indicators suggests that drought stress had already caused structural damage to the photosynthetic organs (Pereira et al., 2000).

In this study, as the duration of drought stress increased, both SWC and LWC progressively decreased. The reduction in LWC led to an increase in leaf temperature, thereby reducing the activity of chloroplasts and Rubisco, which ultimately resulted in a decline in photosynthetic capacity (Yu et al., 2009). During 21-28 days of drought stress, the LWC decreased to 68.5%-72.9%, During this period, a sudden increase in Ci and a sharp decrease in Ls were observed, along with opposing trends in Ci and Cond. This indicates that the LWC range between 68.5% and 72.9% represents the critical threshold for *C. bungei's* transition from SL to NSL. When LWC drops below this threshold, even if the drought is alleviated, it becomes challenging for *C. bungei* to restore its original photosynthetic capacity. This critical LWC range is similar to that observed in maize (73.2%-75.7%) (Song et al., 2020). Interestingly, the fitted curve for LWC/SWC showed a rapid decline in LWC after 14 days of drought stress, once SWC fell below 27.3%. This suggests that when SWC decreases to below 28%, the imbalance between water uptake and transpiration loss intensifies, resulting in a sharp decline in LWC. After 21 days of drought stress, when LWC dropped to approximately 73%, the photosynthesis of *C. bungei* began to transition from SL to NSL, and then the damage to the photosynthetic system gradually worsened and became apparent. Therefore, it is critical to provide timely irrigation before SWC falls below approximately 22% and LWC drops to 73%, to prevent irreversible damage caused by drought.

### 4.2 The synergistic effect of osmotic regulation and antioxidation under prolonged drought stress

Under drought stress, plants not only close stomata to reduce transpiration and minimize water loss but also utilize antioxidant enzymes and non-enzymatic antioxidants synergistically to maintain the dynamic balance of cellular redox status, thereby alleviating drought damage (Gao et al., 2020; Fang and Xiong, 2015). Under mild drought stress, plants can adjust osmotically to lower water potential and maintain turgor. Small organic molecules such as Pro and betaine possess strong osmotic regulation functions and do not disrupt the structure and function of biological macromolecules, making them ideal osmotic substances. In this study, during 7-14 days of drought stress, the expression of *CbP5CS* was significantly upregulated, and the Pro content increased rapidly, playing its role in osmotic regulation to alleviate drought stress. As drought stress intensified and shifted from SL to NSL, both the expression of *CbP5CS* and Pro content showed no significant changes, indicating that osmotic regulation has a limited effect in alleviating severe drought stress. Throughout the experiment, the expression of *CbOAT* showed no significant variation, suggesting that under drought stress, *C. bungei* primarily synthesises Pro *via* the glutamate pathway. In conditions of severe drought, the sucrose content in plant cells increases rapidly, which has a protective effect on the cells. SS content in *C. bungei* leaves showed a significant rise only after 21 days of drought stress. Subsequently, expression levels of *CbSuS* and *CbTPS* remained consistently high to ensure stable synthesis of SS such as sucrose and trehalose. Accumulation of sugars can generate supersaturated solutions with solid mechanical properties, preventing cell solution crystallization and maintaining cells in a stable quiescent state. This is analogous to findings in *Haloxylon ammodendron* (C. A. Mey.) Bunge (Lü et al., 2019), *Adonis amurensis* Regel & Radde (Gao et al., 2020), and other plants. Moreover, disaccharides such as sucrose and trehalose also play roles in stabilizing enzyme activity and protecting membrane structures. The results above indicate that, during different stages of drought stress, the primary osmotic regulatory substances in *C. bungei* vary. Under mild and moderate stress, Pro serves as the main osmotic regulator, whereas as the stress intensifies, SS content gradually become dominant. These substances exhibit a critical LWC threshold at which their concentrations increase sharply, measured at 79% for Pro content and 73% for SS content.

During the period of drought stress from 0 to 14 days, there were no significant changes in SOD enzyme activity, while POD and CAT enzyme activities were significantly reduced. These findings are consistent with the results of Ji et al. (2014). This can be attributed to the dynamic interplay between changes in antioxidant enzyme activity and osmotic regulation. When drought stress is not severe, osmotic regulation plays a major role (Lü et al., 2019), leading to a decreased demand for antioxidant enzymes, especially POD and CAT, which are more sensitive to this response. The expression levels of *CbPOD* and *CbCAT* are both downregulated. However, with the increase in stress duration, from 14 to 21 days of drought, the activities of SOD, POD, and CAT increased by 12.6%, 27.4%, and 194.7%, respectively. This rise is attributed to the accumulation of a significant amount of ROS, which severely disrupts normal plant metabolism (Kekeç et al., 2013). At this stage, the antioxidant enzyme system must work in conjunction with osmotic regulation to maintain cellular homeostasis. SOD rapidly converts O_2_^·-^ into H_2_O_2_ and O_2_, while POD and CAT promptly decompose H_2_O_2_ into H_2_O and O_2_ (Ahmad et al., 2012), thereby reducing the toxic effects of ROS on plants. By day 28 of the stress period, SOD and CAT activities had increased by 21.47% and 74.90%, respectively, compared to day 0, while POD activity remained below the levels that of day 0 of drought. However, it should be noted that the expression of *CbGPX* continues to upregulate under drought stress, indicating that the *C. bungei* may preferentially utilize glutathione peroxidase to degrade peroxides, which may be the reason for the low POD activity. From this, we infer that antioxidant enzymes mainly play a role in the *C. bungei* under severe drought stress, with the activity of CAT showing the most significant changes. The activity of antioxidant enzymes increases sharply between 79% and 73% in LWC, indicating that a large amount of toxic substances such as ROS and H_2_O_2_ accumulate in cells before the transition from SL to NSL. Antioxidant enzymes can alleviate structural damage to photosynthetic organs by increasing their activity.

### 4.3 Coordination of ABA and JA in the drought stress response of *C. bungei*

Plant hormones play a wide and crucial role in plant stress responses (Peleg and Blumwald, 2011). Drought stress induces the accumulation of ABA, and the expression of *CbNCED* continuously increases with the duration of drought stress, stabilizing after 21 days. ABA activates Ca^2+^ channels on the plasma membrane of guard cells, leading to an influx of extracellular Ca^2+^ and an increase in cytosolic Ca^2+^, which in turn activates K^+^ efflux channels, inhibits K^+^ influx channels and plasma membrane K^+^-ATPase activity. This results in a decrease in guard cell osmotic potential and consequently leads to stomatal closure (Liu et al., 2004). ABA can trigger the production of ROS and H_2_O_2_ (Halliwell and Gutteridge, 2015). H_2_O_2_ further activates Ca^2+^ channels, and the resulting Ca^2+^ signal activates the protein kinase OXI1 (oxidative signal-induced kinase 1), which in turn activates the MAPK cascade pathway in response to drought stress. ABA also induces the production of NO, which activates MAPK to induce the upregulation of SOD, CAT, and APX antioxidant enzyme genes and their activities (Zhang et al., 2007). In this study, the content of ABA was consistent with the changes in antioxidant enzyme activity, indicating that ABA enhances the rate of radical scavenging in *C. bungei*. In this study, the trends in JA content and *CbOPR* expression were consistent with those of ABA, both increasing with drought stress and showing a significant upregulation by day 14. JA is an important stress signal substance in adversity stress, widely present in plants. When JA accumulates to a certain level, it triggers,some defense genes will be activated for expression (He et al., 2015)。Studies on tobacco leaves under high-temperature stress have shown that JA treatment enhances the activity of leaf antioxidant enzymes, reduces osmotic potential, and lowers soluble protein content, thereby alleviating stress imposed on growth (Yang et al., 2014). Under drought stress, JA treatment in rice leaves resulted in increased water potential, decreased SS and Pro content, and enhanced the rice plant’s ability to cope with drought stress (Zhang et al., 2011). Although JA can alleviate drought stress, studies also indicate that JA can promote premature senescence and aging in plants. Ge et al (2007) suggests that JA induces senescence in *Ginkgo biloba* leaves to reduce transpirational water loss as a strategy against adversity. JA and ABA may potentially coordinate in some manner under drought stress conditions. Wang et al. (2020) found that the ABA and JA signaling networks play crucial roles in the induction of drought tolerance through drought conditioning, with JA upstream regulating ABA in the signaling network, collectively mediating the formation of drought tolerance. The transcription factor NaWRKY70 plays a crucial role in regulating the biosynthesis of JA-Ile and ABA, thereby coordinating their functions in plant stress resistance (Song and Wu, 2024). Under drought stress, when LWC falls below 79% by the 14th day, the levels of ABA and JA significantly increase, working together through the aforementioned mechanisms to combat drought stress. However, following the transition from SL to NSL (drought stress between days 21-28), the increase in ABA and JA levels is no longer significant. At this stage, reliance on free radical scavenging or reducing transpirational water loss is insufficient to alleviate the damage caused by drought stress in *C. bungei*.

## 5 Conclusions

**Figure 9:**
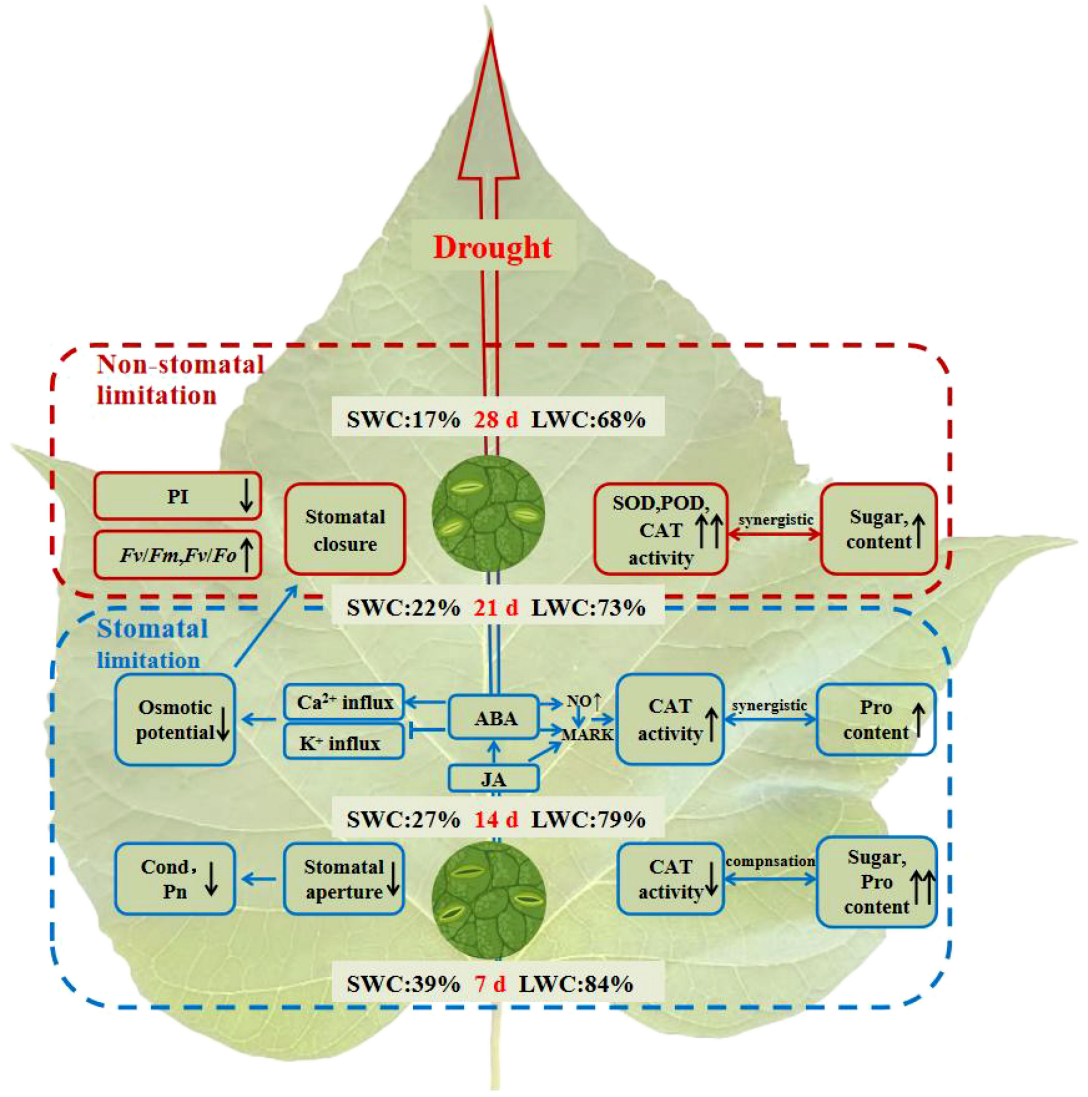
Diagram illustrating the response of *C. bungei* to prolonged drought stress. Under mild drought stress (7-14 days), *C. bungei* reduce stomatal conductance (Cond) to minimize water loss, resulting in decreased intercellular CO_2_ concentration (Ci) and photosynthetic rate (Pn). Increased levels of proline (Pro) and soluble sugars (SS) content enhance osmotic regulation. Antioxidant enzyme activity complements osmotic regulation dynamics, significantly reducing catalase (CAT) activity. Under moderate drought stress (14-21 days), accumulation of ABA promotes extracellular Ca^2+^ influx, inhibits K^+^ influx, reduces guard cell turgor pressure, and further closes stomata. JA and ABA synergistically activate MAPK cascade reactions, enhancing CAT activity, and jointly responding to drought stress with osmotic regulation (Pro). Under severe drought stress (21-28 days, SWC22%, LWC73%), the dynamic equilibrium between reactive oxygen species production and scavenging is completely disrupted. Antioxidant enzyme activity and soluble sugar content further increase. Structural damage to photosynthetic organs occurs, shifting from stomatal to non-stomatal limitation. At this stage, reducing transpiration, scavenging free radicals, and osmotic regulation no longer suffice to alleviate drought-induced damage to *C. bungei*.

The *C. bungei* mitigates mild drought stress (7-14 days of drought stress) through osmotic regulation (**Figure 9**). As drought stress persists (14-21 days of drought stress), abscisic acid (ABA) and jasmonic acid (JA) coordinate antioxidant and osmotic regulation. However, under severe drought stress (21-28 days of drought stress), characterized by a SWC reduction to 22% and a LWC decrease to 73%, damage to the photosynthetic system occurs, and SL changed to NSL. The ability to reduce transpiration, eliminate free radicals, and regulate osmotic pressure becomes insufficient to alleviate the injury caused by drought stress on *C. bungei*.

## Data Availability

The original contributions presented in the study are included in the article/supplementary material. Further inquiries can be directed to the corresponding authors.
